# Cecal Patches Generate Abundant IgG2b-Bearing B Cells That Are Reactive to Commensal Microbiota

**DOI:** 10.1155/2022/3974141

**Published:** 2022-05-04

**Authors:** Masato Tsuda, Hiraku Okada, Natsuki Kojima, Fumiya Ishihama, Yuhei Muraki, Toshiki Oguma, Nanako Hattori, Takumi Mizoguchi, Kiyoaki Mori, Satoshi Hachimura, Yoshimasa Takahashi, Kyoko Takahashi, Shuichi Kaminogawa, Akira Hosono

**Affiliations:** ^1^Department of Food Bioscience and Biotechnology, College of Bioresource Sciences, Nihon University, 1866 Kameino Fujisawa-shi, Kanagawa 252-0880, Japan; ^2^Research Center for Food Safety, Graduate School of Agricultural and Life Sciences, The University of Tokyo, 1-1-1 Yayoi, Bunkyo-ku, Tokyo 113-8657, Japan; ^3^Research Center for Drug and Vaccine Development, National Institute of Infectious Diseases, 1-23-1 Toyama, Shinjuku-ku, Tokyo 162-8640, Japan; ^4^Department of Applied Biological Science, College of Bioresource Sciences, Nihon University, 1866 Kameino Fujisawa-shi, Kanagawa 252-0880, Japan

## Abstract

Gut-associated lymphoid tissue (GALT), such as Peyer's patches (PPs), are key inductive sites that generate IgA^+^ B cells, mainly through germinal center (GC) responses. The generation of IgA^+^ B cells is promoted by the presence of gut microbiota and dietary antigens. However, the function of GALT in the large intestine, such as cecal patches (CePs) and colonic patches (CoPs), and their regulatory mechanisms remain largely unknown. In this study, we demonstrate that the CePs possess more IgG2b^+^ B cells and have fewer IgA^+^ B cells than those in PPs from BALB/c mice with normal gut microbiota. Gene expression analysis of postswitched transcripts supported the differential expression of dominant antibody isotypes in B cells in GALT. Germ-free (GF) mice showed diminished GC B cells and had few IgA^+^ or IgG2b^+^ switched B cells in both the small and large intestinal GALT. In contrast, myeloid differentiation factor 88- (MyD88-) deficient mice exhibited decreased GC B cells and presented with reduced numbers of IgG2b^+^ B cells in CePs but not in PPs. Using ex vivo cell culture, we showed that CePs have a greater capacity to produce total and microbiota-reactive IgG2b, in addition to microbiota-reactive IgA, than the PPs. In line with the frequency of GC B cells and IgG2b^+^ B cells in CePs, there was a decrease in the levels of microbiota-reactive IgG2b and IgA in the serum of GF and MyD88-deficient mice. These data suggest that CePs have a different antibody production profile compared to PPs. Furthermore, the innate immune signals derived from gut microbiota are crucial for generating the IgG2b antibodies in CePs.

## 1. Introduction

The immune system of the gut contributes to the maintenance of intestinal homeostasis in the face of continual exposure to large amounts of food-derived antigens, commensal microbiota, and invading pathogens. The gut-associated lymphoid tissue (GALT) is a key inductive site for initiating antigen-specific immune responses against such luminal antigens. GALT is composed of Peyer's patches (PPs), which are located in the small intestine, and their counterparts in the cecum (cecal patches (CePs)) and colon (colonic patches (CoPs)) and solitary intestinal lymphoid tissue, such as the cryptopatches and isolated lymphoid follicles that are present throughout the intestine [1–3]. The primary role of the GALT is the generation of immunoglobulin A- (IgA-) producing B cells [4]. The B cell follicles in PPs contain chronic germinal centers (GCs), a microenvironment where activated B cells expressing activation-induced cytidine deaminase (AID) not only induce class-switch recombination from IgM into IgA but also drive somatic hypermutation to generate antibody diversity [5]. In addition, subepithelial dome (SED) of PPs is reported to be the sites of localization of activated B cells at the pre-GC stage which express AID and undergo class-switch recombination to IgA [6, 7]. IgA^+^ B cells generated in PPs then enter circulation and systemically travel through blood and lymphatics into the intestinal lamina propria. After reaching the lamina propria, the IgA^+^ B cells terminally differentiate into plasma cells to secrete dimeric IgA. In addition, follicular helper T (Tfh) cells and follicular dendritic cells (FDCs) directly interact with B cells in the GCs of PPs to promote these reactions and mediate the selection of B cells expressing high-affinity antibodies [8]. Dysregulation of selection of IgA-producing B cells in GCs of PPs results in alterations in the composition of gut microbiota [9]. However, the function of GALT in the large intestine has not been well described. It is likely that the GALTs in the large intestine, namely, CePs and CoPs, are responsible for the induction of IgA^+^ B cells with different migratory properties. Adoptive transfer experiments have shown that IgA^+^ B cells derived from CePs migrate to both the large and small intestines, whereas B cells from the PPs are preferentially recruited to the small intestine [1]. It has been suggested that CoPs are the main sites for the induction of antigen-specific IgA-producing cells, which migrate into the large intestine in response to intrarectal immunization and/or following systemic priming [10]. However, it is unknown whether there are differences in the ability of the GALTs in the small and large intestines to generate IgA^+^ B cells, switch to other antibody isotypes, or produce antibodies with different affinities.

In addition, the environmental factors in the gut largely influence the development of GCs in PPs. In mice maintained under normal breeding conditions, it has been observed that unlike systemic lymphoid organs, the GCs in the PPs are continuously induced due to constant stimulation by the gut microbiota and food-derived antigens [4, 11, 12]. Moreover, germ-free (GF) mice or mice treated with antibiotics (to reduce and/or eliminate gut microbiota) have fewer GCs in their PPs [13, 14]. In particular, it has been reported that defects in signaling via myeloid differentiation factor 88 (MyD88), which is an adaptor protein for most toll-like receptors (TLRs), plays a key role in microbiota-dependent signaling, leading to poor GC responses and development of IgA^+^ B cells in the PPs [15]. In addition, it has been reported that the mice fed on an antigen-free diet, which is a chemically defined elemental diet, show decreased GC formation in PPs, compared to the mice on a normal diet under either specific-pathogen free (SPF) or GF condition [11, 12]. Although it is known that the abundance and diversity of the commensal bacteria and food-derived antigens in the lumen vary along the different sections of the gastrointestinal tract, it remains to be elucidated whether GC formation and generation of class-switched antibody isotypes are differentially regulated in the GALTs of the small and large intestines.

In this study, we demonstrated that the GALTs in the cecum, i.e., CePs, have more IgG2b^+^ B cells and fewer IgA^+^ B cells than those in the PPs. Furthermore, compared to the PPs, CePs produced increased levels of microbiota-reactive IgG2b and IgA. Although intestinal microbiota is essential for inducing GC responses that generate the IgA and IgG2b antibody isotypes in the GALTs of both the small and large intestines, gut microbiota-dependent innate immune signaling is crucial for these responses in CePs. These signaling pathways also contribute to the supply of microbiota-reactive IgA and IgG2b in systemic circulation in correlation with the abundance of GC responses in CePs. These data provide a novel role for CePs in antibody production.

## 2. Materials and Methods

### 2.1. Mice

Wild-type (WT) BALB/c mice were purchased from CLEA Japan (Tokyo, Japan) and maintained under either conventional (CV) or SPF conditions. MyD88^−/−^ mice on a BALB/c background were purchased from Oriental BioService (Kyoto, Japan) and kept under SPF conditions. GF BALB/c mice were maintained in sterile vinyl isolators at an animal facility. GF conditions of the isolators were verified every month based on the aerobic and anaerobic cultures and gram staining of fresh feces from the mice. Mice were maintained at 23–25°C and 40–60% humidity and under a 12 : 12 h light-dark cycle. Eight- to fourteen-week-old mice were used for this study.

### 2.2. Preparation of Serum and Fecal Samples

Serum samples were prepared by centrifuging (3000 rpm for 10 min at 4°C) whole blood samples that were collected via cardiac puncture. To prepare fecal extracts, freshly collected feces were suspended and homogenized in PBS containing 50 mM ethylenediaminetetraacetic acid (EDTA) and 0.1 mg/ml trypsin inhibitor (Sigma-Aldrich, St. Louis, MO, USA). These extracts were adjusted to a concentration of 25 mg/ml of feces. The supernatants were collected after stepwise centrifugation with increasing force (3000 rpm for 10 min, 10000 rpm for 10 min, and 15000 rpm for 10 min at 4°C) of the homogenates.

### 2.3. Cell Preparation

Single cells were prepared from the GALTs from small and large intestines using the method described previously [16]. Briefly, PPs, CePs, and CoPs were washed with RPMI 1640 medium (Nissui, Tokyo, Japan) supplemented with 50 U/ml penicillin, 50 *μ*g/ml streptomycin, 2 mM L-glutamine, and 50 *μ*M 2-mercaptoethanol, minced into small pieces, and then incubated in RPMI 1640 medium containing 10% FCS (Biowest, Nuaillé, France), 1 mg/ml collagenase D (Roche Diagnostics, Mannheim, Germany) (Roche Diagnostics, Indianapolis, IN, USA), and 20 *μ*g/ml DNase I (Roche Diagnostics) for 50 min. The cell suspensions were filtered through a 70 *μ*m cell strainer, washed, and resuspended in RPMI 1640 medium containing 10% FCS.

### 2.4. Cell Culture

Single-cell preparations (between 1 × 10^5^ cells and 5 × 10^5^ cells) from the small or large intestinal GALT were cultured in 96-well flat-bottom plates in the presence of lipopolysaccharide (LPS, extracted from *Escherichia coli* O55:B5, Sigma-Aldrich), Pam3CSK4 (InvivoGen, San Diego, CA, USA), or CpG DNA (ODN 1826, InvivoGen) for 7 days. The supernatants of the cell cultures were collected and used for the enzyme-linked immunosorbent assay (ELISA).

### 2.5. Flow Cytometry

Freshly isolated cells were washed in Dulbecco's modified Eagle's medium (DMEM, Sigma-Aldrich) supplemented with 1% bovine serum albumin (Sigma-Aldrich), 10 mM HEPES, 2 mM sodium pyruvate, 10^−3^ mM EDTA, and 0.01% NaN_3_. Nonspecific binding to Fc receptors was blocked with anti-CD16/CD32 (93; BioLegend, San Diego, CA, USA) for 15 min. Cells were treated with biotin-conjugated antibodies and then incubated for 20 min on ice. The cells were washed twice with supplemented DMEM. Fluorescence-conjugated antibodies or streptavidin was added to the cells and incubated for 20 min on ice. The cells were washed and resuspended in supplemented DMEM. Flow cytometry was performed using FACSCanto (BD Biosciences, Franklin Lakes, NJ, USA), and data analysis was performed using FlowJo software v10.3 (Tree Star, Ashland, OR, USA).

The following biotin or fluorescence-labeled antibodies were used: biotin anti-IgM (II/41; eBioscience, San Diego, CA, USA), biotin anti-IgA (11-44-2; eBioscience), biotin anti-IgG1 (A85-1; BD Biosciences), biotin anti-IgG2b (R12-3; BD Biosciences), biotin anti-IgG3 (R40-82; BD Biosciences), biotin anti-CXCR5 (SPRCL5; eBioscience), biotin rat IgG1 isotype control (eBRG1; eBioscience), biotin rat IgG2a isotype control (eBR2a; eBioscience), fluorescein isothiocyanate (FITC) anti-CD4 (RM4-5), PerCPCyanin5.5 anti-mouse CD3*ε* (145-2C11, BioLegend), APC (allophycocyanin) anti-programmed cell death protein 1 (PD-1) (RMP1-30; BioLegend), FITC anti-B220 (RA3-6B2; BioLegend), PE anti-GL7 (GL-7; BD Biosciences), Alexa Fluor647 anti-CD95 (Jo2; BD Biosciences), PerCPCyanin5.5 anti-mouse CD38 (90; BioLegend), PerCPCyanin5.5 anti-mouse CD196 (CCR6, 29-2L17; BioLegend), PerCPCyanin5.5 rat IgG2a isotype control (R35-95; BD Biosciences), PerCPCyanin5.5 Armenian hamster IgG isotype control (HTK888; BioLegend), and streptavidin-PE and streptavidin-PECyanin7 (eBioscience).

### 2.6. ELISA

Concentrations of total IgA, IgG2b, and IgM in culture supernatants, serum, and fecal extracts were determined by sandwich ELISA, with minor modifications to protocol described previously [17]. Goat anti-mouse IgG F(ab′)2 fragment antibody (Sigma-Aldrich) was used as a capture antibody to measure all antibody isotypes. Alkaline phosphatase-conjugated goat anti-mouse IgA, IgG2b, and IgM (Southern Biotech, Birmingham, AL, USA) were used to detect the antibodies in the collected samples. Color development was performed by adding p-nitrophenyl phosphate (Fujifilm Wako Pure Chemical Corporation, Osaka, Japan) as the substrate. Optical density was measured at 405 nm using a Multiskan GO microplate spectrophotometer (Thermo Fisher Scientific, Waltham, MA, USA).

The amount of microbiota-reactive IgA and IgG2b was measured using the bacteria isolated from fecal samples of CV BALB/c mice (instead of capture antibodies) and as per the procedure described previously [18]. Briefly, fecal bacteria were prepared from 0.2 g of freshly isolated feces homogenized in sterile PBS, and the pellet containing debris and mouse cells was removed after centrifugation at 1000 rpm for 5 min, followed by filtration through a 40 *μ*m cell strainer. The isolated bacteria were washed twice with PBS, heat-killed at 85°C for 1 h, and then resuspended in 10 ml of 0.05 M carbonate-bicarbonate buffer (pH 9.6). Fecal bacterial preparations (100 *μ*l) were added to the wells of an ELISA plate. Subsequently, the above-mentioned procedures were repeated to measure total IgA and IgG2b levels.

### 2.7. Quantitative Reverse Transcription Polymerase Chain Reaction (RT-PCR)

Total RNA was isolated from single cells of small and large intestinal GALT using a High Pure RNA Isolation Kit (Roche Diagnostics, Mannheim, Germany) and reverse transcribed into cDNA using SuperScript™ III Reverse Transcriptase (Invitrogen, Carlsbad, CA). Quantitative PCR was performed using the LightCycler® 96 system (Roche Diagnostics, Mannheim, Germany) and the KAPA SYBR Fast qPCR kit (Kapa Biosystems Inc., Wilmington, MA, USA). Thermal cycling conditions were as follows: 95°C for 3 min for preincubation, 45 cycles at 95°C for 10 s, 63°C for 20 s, and 72°C for 1 s. Data were analyzed using the LightCycler 96 software version 1.1 (Roche Diagnostics). mRNA expression of target genes was normalized to the expression of *hypoxanthine phosphoribosyltransferase* (*Hprt*) as an internal control, and the relative gene expression was calculated using the 2^−ΔΔCt^ method.

Primers used for *Hprt*, *Aicda*, and post-class-switched transcript (PST) for IgA and IgG2b antibodies are as follows: *Hprt*—forward: 5′-GCCCCAAAATGGTTAAGGTT-3′ and reverse: 5 ′-TTGCGCTCATCTTAGGCTTT-3′; *Aicda*—forward: 5′-AAATTCTGTCCGGCTAACCA-3′ and reverse: 5′-CATTCCAGGAGGTTGCTTTC-3′; PST*α*—forward: 5′-ACCTGGGAATGTATGGTTGTGGCTT-3′ and reverse: 5′-GAGCTGGTGGGAGTGTCAGTG-3′; and PST*γ*2b—forward: 5′-ACCTGGGAATGTATGGTTGTGGCTT-3′ and reverse: 5′-CGGAGGAACCAGTTGTATC-3′.

### 2.8. Statistical Analysis

Statistical significance was calculated using the SPSS software (IBM SPSS Statistics 20, International Business Machines Corp., Armonk, NY, USA). Data were analyzed using one-way analysis of variance (ANOVA) followed by Tukey's HSD test or Dunnett's T3 test. The unpaired two-tailed Student's *t*-test was used for analyzing the serum and fecal antibody concentrations. Differences were considered statistically significant at *p* < 0.05.

## 3. Results

### 3.1. Effect of Commensal Microbiota on GC Formation and Cell Surface Antibody Expression on B Cells in the GALTs of the Small and Large Intestines

**Figure 1:**
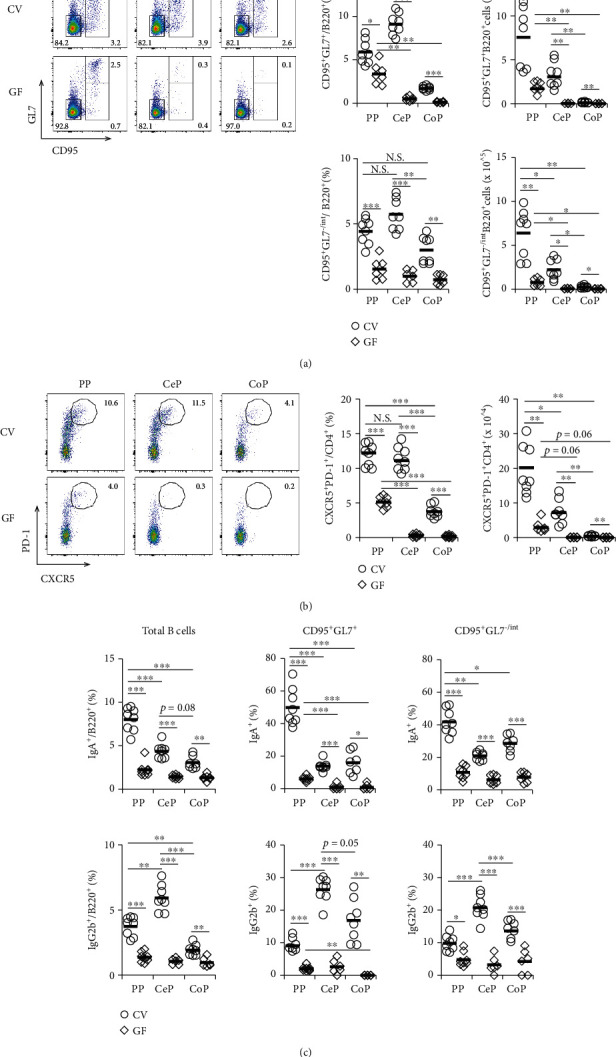
The role of gut microbiota in GC formation and antibody expression on B cells in the GALT of the small and large intestines. Flow cytometric analysis of mononuclear cells of GALT in CV and GF mice was performed. (a) Left panel: flow cytometric dot plot of CD95 and GL7 expression in B220^+^ cells. Right panel: percentage and absolute numbers of CD95^+^GL7^+^B220^+^ GC B cells and CD95^+^GL7^-/int^B220^+^ pre-GC B cells. (b) Left panel: flow cytometric dot plot of CXCR5 and PD-1 expression in CD4^+^CD3^+^ cells. Right panel: percentage and absolute numbers of CXCR5^+^PD-1^+^CD4^+^CD3^+^ Tfh cells. (c) The percentage of B220^+^ total B cells, CD95^+^GL7^+^B220^+^ GC B cells, and CD95^+^GL7^-/int^B220^+^ pre-GC B cells expressing IgA or IgG2b. Data are shown as the mean ± SD and are representative of flow cytometric dot plots from six to eight independent experiments. ^∗^*p* < 0.05, ^∗∗^*p* < 0.01, and ^∗∗∗^*p* < 0.001.

To determine the difference in the formation of GCs in GALT of the small and large intestines, we analyzed the cellularity of GC B cells and Tfh cells in CV BALB/c mice. The frequency of CD95^+^GL7^+^B220^+^ GC B cells and CXCR5^+^PD-1^+^CD4^+^CD3^+^ Tfh cells in PPs and CePs was higher than that in CoPs (Figures 1(a) and 1(b), Figure S1a). The absolute numbers of these cells were highest in PPs, intermediate in CePs, and lowest in CoPs and might be dependent on the total cellularity of each tissue (Figures 1(a) and 1(b)). Furthermore, a similar trend was found in the frequency and absolute number of the CD95^+^GL7^-/int^B220^+^ B cell subpopulation in each GALT (Figure 1(a)). Then, we tried to characterize this B cell faction and found that these B cells included chemokine receptor 6 (CCR6)^+^ and CD38^+^ populations (Figure S2), representing the pre-GC stage [6, 19, 20]. Next, we analyzed the expression of antibody isotypes on each B cell subpopulation in GALT. The expression of IgA on total B cells was higher in PPs than in the GALT from the large intestine (Figure 1(c) and Figure S1b). We then investigated the levels of the other isotypes that are highly expressed by B cells in the GALT of the large intestine and found that the expression of IgG2b on total B cells was highest in CePs (Figure 1(c) and Figures S1c and S3). These differential expression patterns of antibody isotypes were more prominent in not only GC but also pre-GC B cells. While the B cell populations from PPs preferentially expressed IgA, those from CePs predominantly expressed IgG2b (Figure 1(c), Figure S1b and c).

Thereafter, we investigated the impact of the commensal microbiota on GC formation and cell surface antibody expression in B cells from the GALT in the small and large intestines of GF mice compared to CV mice. The proportion and absolute numbers of GC B cells, pre-GC B cells, and Tfh cells in each tissue were significantly reduced in GF mice (Figures 1(a) and 1(b)). We detected fewer GC B cells and Tfh cells within CePs and CoPs than in the PPs of GF mice. Furthermore, the expression of both IgA and IgG2b on total, pre-GC, and GC B cells in each tissue was dramatically reduced in GF mice compared to CV mice (Figure 1(c)).

Taken together, these data suggest that compared to the B cells in PPs, which are IgA^+^ B cells generated in a commensal microbiota-dependent manner, those in CePs are IgG2b^+^ B cells.

### 3.2. Differential Class-Switch Recombination in GALT from the Small and Large Intestines

**Figure 2:**
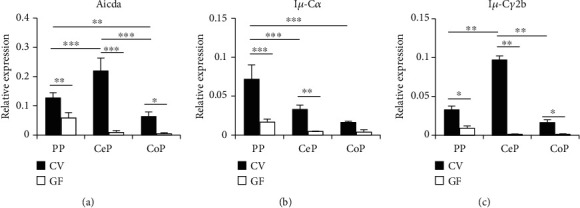
Analysis of gene expression to evaluate class-switch recombination in the GALT from small and large intestines. Total RNA was isolated from mononuclear cells from the GALT in CV and GF mice. The gene expression of *Aicda* and postswitched transcripts for IgA and IgG2b was measured by real-time PCR. Data are shown as the mean ± SD for three to five independent experiments. ^∗^*p* < 0.05, ^∗∗^*p* < 0.01, and ^∗∗∗^*p* < 0.001.

To further characterize the differential expression of the dominant antibody isotype on B cells from the GALT in small and large intestines, we examined the process of class-switch recombination. After evaluating the relative proportion of B220^+^ B cells in each tissue (data not shown), we analyzed the expression of the transcripts for AID (*Aicda*), which is an indispensable enzyme for class-switch recombination and that of the post-class-switched transcripts for IgA and IgG2b in mononuclear cells from CV and GF mice. We observed that the expression of *Aicda* was higher in PPs and CePs than in the CoPs of CV mice (Figure 2(a)). These expression patterns are consistent with the frequency of GC B cells in each tissue. The expression of *Iμ-Cα*, a rearranged transcript for IgA, was highest in PPs than in the GALTs in the large intestine (Figure 2(b)). In contrast, we observed the highest expression of *Iμ-Cγ2b*, a postswitched transcript for IgG2b, in CePs compared to other GALTs (Figure 2(c)). The expression of *Aicda* and that of the postswitched transcripts for both IgA and IgG2b were markedly lower in GF mice than in CV mice. These data suggest that B cells from the GALT in the small and large intestines show differential class-switch recombination.

### 3.3. Function of MyD88 Signaling on GC Formation and Cell Surface Antibody Expression in B Cells from the GALT of the Small and Large Intestines

**Figure 3:**
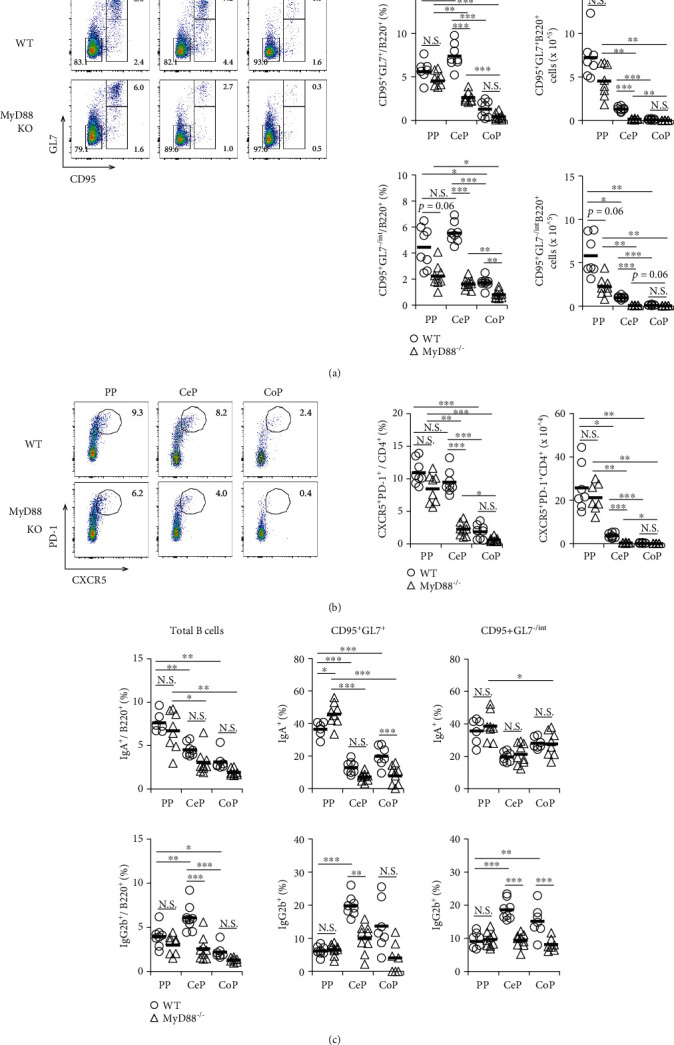
The role of MyD88 signaling in GC formation and antibody expression on B cells in GALT from the small and large intestines. Flow cytometric analysis of the mononuclear cells of each GALT from SPF WT and MyD88^−/−^ mice was performed. (a) Left panel: flow cytometric dot plot of CD95 and GL7 expression in B220^+^ cells. Right panel: percentage and absolute numbers of CD95^+^GL7^+^B220^+^ GC B cells and CD95^+^GL7^-/int^B220^+^ pre-GC B cells. (b) Left panel: flow cytometric dot plot of CXCR5 and PD-1 expression in CD4^+^CD3^+^ cells. Right panel: percentage and absolute numbers of CXCR5^+^PD-1^+^CD4^+^CD3^+^ Tfh cells. (c) The percentage of B220^+^ total B cells, CD95^+^GL7^+^B220^+^ GC B cells, and CD95^+^GL7^-/int^B220^+^ pre-GC B cells expressing IgA or IgG2b. Data are shown as mean ± SD and are representative of the flow cytometric dot plot from seven to eight independent experiments. ^∗^*p* < 0.05, ^∗∗^*p* < 0.01, and ^∗∗∗^*p* < 0.001.

MyD88 is a key adaptor molecule in the TLR signaling cascade, which is triggered in response to the gut microbiota. To examine the role of MyD88 in the development of GCs and the expression of antibody isotypes on B cells from the GALT of the small and large intestines, we used the MyD88^−/−^ mice and compared them with WT BALB/c mice kept under SPF condition. We found that the proportion and absolute numbers of GC B cells and Tfh cells were significantly reduced in CePs of MyD88^−/−^ mice (Figures 3(a) and 3(b)). Although the proportion and absolute numbers of GC B cells and Tfh cells in the PPs and CoPs of MyD88^−/−^ mice were slightly lower than those of WT mice, the difference was not statistically significant. IgA expression on total B cells in each tissue was comparable between MyD88^−/−^ and WT mice (Figure 3(c)). However, IgG2b expression in total, pre-GC, and GC B cells in CePs was significantly reduced in MyD88^−/−^ mice (Figure 3(c)). IgG2b expression in pre-GC B cells from CoPs was also significantly reduced in MyD88^−/−^ mice (Figure 3(c)). However, compared to WT mice, there was no decrease in IgA expression in GC and pre-GC B cells from PPs of MyD88^−/−^ mice (Figure 3(c)). We noticed that IgA expression in GC B cells from CoPs was significantly reduced and that in the GC B cells of CePs from MyD88^−/−^ mice had a decrease tendency compared to that of WT mice (Figure 3(c)). These data suggest that MyD88 signaling is essential for GC formation and IgG2b expression on B cells within CePs and contributes to the induction of IgA expression in GC B cells in the GALT of the large intestine.

### 3.4. B Cells in CePs Produce High Levels of Commensal Bacteria-Reactive IgG2b in Response to TLR Ligands

**Figure 4:**
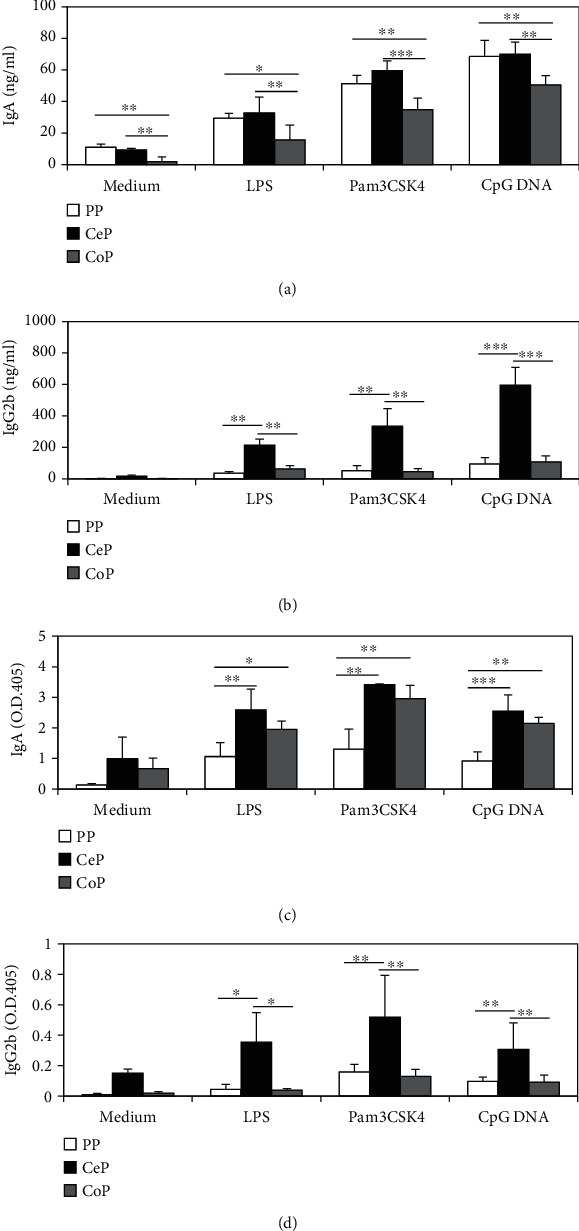
Production of the different antibody isotypes by GALT cells from small and large intestines. Mononuclear cells from GALT in CV BALB/c mice were stimulated *in vitro* with 1 *μ*g/ml LPS, 1 *μ*g/ml Pam3CSK4, or 1 *μ*M of CpG DNA for 7 days. (a, b) The concentrations of total IgA and IgG2b in the culture supernatants were measured by ELISA. Data are shown as mean ± SD. (c, d) The levels of fecal bacteria-reactive IgA and IgG2b in the culture supernatants were measured by ELISA. Antibody titers of fecal bacteria-reactive antibodies are shown as net OD405 units (sample OD405–mean OD405 of blanks). The mean OD405 value of blanks was 0.21 for IgA and 0.07 for IgG2b. The results are representative of at least three independent experiments. ^∗^*p* < 0.05, ^∗∗^*p* < 0.01, and ^∗∗∗^*p* < 0.001.

Next, we examined the ability of GALTs in the large intestine to produce class-switched isotypes of antibodies and evaluated the antigen specificity of these antibodies. We isolated single cells from the GALT in CV BALB/c mice and stimulated the isolated cells with several ligands for TLRs, such as LPS, Pam3CSK4, and CpG DNA, which can not only stimulate B cells to induce class-switch recombination and antibody production but also activate dendritic cells and T cells to help the antibody production by B cells [21]. The production of total IgA in response to TLR ligands was comparable between PPs and CePs and lower in CoPs (Figure 4(a)). In contrast, we found that the production of total IgG2b was highest in the CePs (Figure 4(b)).

Since PPs are important sites for generating IgA against gut microbiota, we next examined the levels of antibodies that were capable of binding fecal bacteria. Mononuclear cells from the GALT in the large intestine were able to produce higher levels of commensal microbiota-reactive IgA than the cells from PPs (Figure 4(c)). Therefore, this indicates that compared to the GALT from the large intestine, PPs have a higher population of IgA-producing cells with a specificity other than that for commensal microbiota. Additionally, we found that the production of commensal bacteria-reactive IgG2b was highest for the mononuclear cells from CePs (Figure 4(d)). Collectively, these findings suggest that CePs could be important for generating commensal bacteria-reactive IgG2b antibodies and that compared to PPs, the GALT from the large intestine generates increased levels of commensal bacteria-reactive IgA in response to TLR ligands.

### 3.5. Commensal Microbiota-Dependent MyD88 Signaling Is Crucial for the Production of Commensal Microbiota-Reactive Antibodies in Systemic Circulation

**Figure 5:**
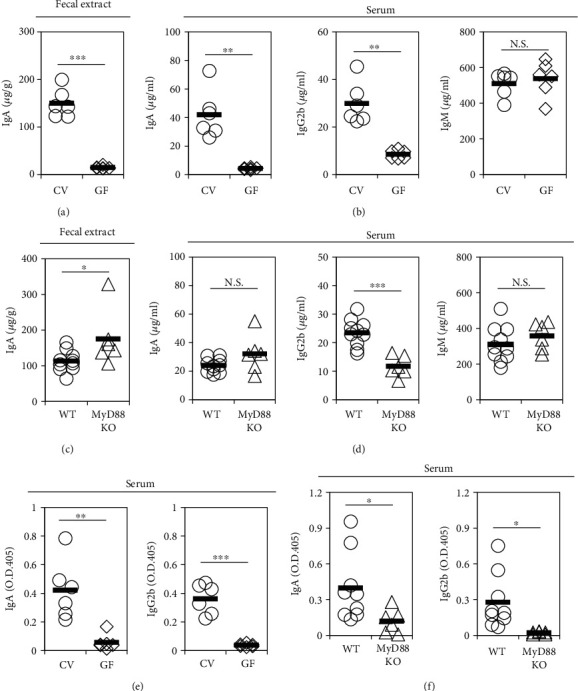
Levels of total and commensal microbiota-reactive antibodies in the feces and serum of the mice maintained under the different experimental conditions. The levels of total or commensal microbiota-reactive antibodies were measured by ELISA. (a) Total IgA in the fecal extract from CV and GF WT mice. (b) Total IgA, IgG2b, and IgM in serum from CV and GF WT mice. (c) Levels of total IgA in the fecal extract from SPF WT and MyD88^−/−^ mice. (d) Total IgA, IgG2b, and IgM in serum from SPF WT and MyD88^−/−^ mice. (e) Commensal microbiota-reactive IgA and IgG2b from CV and GF WT mice. (f) Commensal microbiota-reactive IgA and IgG2b from SPF WT and MyD88^−/−^ mice. (e, f) Antibody titers of commensal microbiota-reactive antibodies are shown as net OD405 units (sample OD405–mean OD405 of blanks). The mean OD405 value of blanks was 0.13 for IgA and 0.06 for IgG2b. Data are shown as the mean ± SD of six to ten mice and are representative of at least three independent experiments. ^∗^*p* < 0.05, ^∗∗^*p* < 0.01, and ^∗∗∗^*p* < 0.001.

Next, we aimed to determine whether the pool of secreted antibodies in the intestinal mucosa and systemic circulation reflected the differential expression patterns of antibody isotypes on B cells from the GALT. Therefore, we examined the levels of total and commensal bacteria-reactive antibodies in the feces and serum of mice maintained under the different experimental conditions. Consistent with previous reports [22], the concentrations of total IgA in the feces (Figure 5(a)) and those of the total IgA and IgG2b in serum were lower in GF mice than in CV mice (Figure 5(b)). In addition, commensal bacteria-reactive IgA and IgG2b levels were also decreased in the serum of GF mice (Figure 5(e)). Unlike GF mice, the concentration of total IgA in fecal extracts (Figure 5(c)) and serum (Figure 5(d)) from SPF MyD88-deficient mice was higher and comparable, respectively, than that from SPF WT mice. However, there was a decrease in commensal-reactive IgA in the serum of MyD88-deficient mice compared to that of SPF WT mice (Figure 5(f)). In contrast, we found that the amount of total and commensal microbiota-reactive serum IgG2b was significantly decreased in MyD88-deficient mice compared to SPF WT mice (Figure 5(f)). These data suggest that the commensal microbiota, via the MyD88 signaling pathway, induces commensal bacteria-reactive IgA and IgG2b in serum; however, it is likely that IgA with specificity for antigens other than the commensal microbiota could be generated in a MyD88 signaling-independent manner.

## 4. Discussion

Ideally, the GALT is known to produce antigen-specific IgA^+^ B cells. However, the role of GALT in generating B cells expressing other isotypes remains unclear. In the present study, we characterized the differential features of GC B cells and those of B cells bearing class-switched isotype of antibodies from the GALT in the large intestine, particularly, in CePs. Specifically, we demonstrated that CePs possess a large population of IgG2b^+^ B cells and compared them to the cells from PPs; those derived from CePs produce higher levels of total and microbiota-reactive IgG2b. Gut microbiota-dependent MyD88 signaling led to the formation of GCs and generation of IgG2b^+^ B cells in CePs.

Previous studies suggested that the gut microbiota contributes to GC formation and the development of Tfh cells in PPs [13, 14]. However, our data suggest that the gut microbiota was largely responsible for mediating these events in the GALT of the large intestine. In addition, MyD88 signaling facilitated the development of GC in CePs but not in PPs. These differences between the GALT in the small and large intestines may be explained in part by the effect of dietary antigens [11, 12], which can also stimulate GC reactions in PPs. Therefore, our data suggests that different quantities and qualities of environmental factors between the small and large intestines regulate the functional development and homeostasis of their own GALT.

Our data revealed that CePs are more abundant in IgG2b^+^ B cells and have fewer IgA^+^ B cells than the PPs in CV and SPF BALB/c mice. The levels of postswitched transcripts for each isotype in the different GALT further support the isotype class switching patterns in B cells. Class switching in B cells is dependent on the several cytokines produced by T cells and dendritic cells [23]. It is well established that transforming growth factor- (TGF-) *β*1 can induce class-switch recombination to both IgA and IgG2b [24]. However, thus far, host-derived or environmental factors that mediate class switching to IgG2b alone have not been identified. Moreover, TGF-*β*1 in combination with IL-5, IL-21, and retinoic acid (RA), which is a vitamin A metabolite, selectively promotes class-switch recombination to IgA but inhibits IgG2b [25–27]. Thus, we speculate that there may be differences in the abundance of these factors between the GALT in the small and large intestine, resulting in the differential expression of preferential class-switched antibody isotypes on B cells. In line with this idea, it has been reported that the concentrations of RA follow a gradient from the proximal to distal intestine, with higher concentrations being found in the proximal small intestine and lower concentrations in the large intestine [28]. Administering a vitamin A-deficient diet induced a decrease in IgA^+^ B cells and GC B cells in PPs [15]. Future studies must identify the specific factors and determine the underlying mechanisms that regulate the IgA/IgG2b balance in each GALT.

GCs in secondary lymphoid tissues are well considered to be key sites where B cells undergo class-switch recombination. On the other hand, the previous report has suggested that IgA class switching was observed within PPs in the absence of GC. In fact, PP B cells represented the pre-GC stage with GL7^int^ phenotype-expressed AID and the markers for IgA class switching [29]. In addition, recent papers have reported that IgA class-switch recombination may occur in pre-GC B cells which express CCR6 to access into SED and interact with dendritic cells and/or M cells before entering the GC [6, 7]. In this context, our data showed that the cell surface expression of IgG2b in addition to IgA was detected in the pre-GC B cell subpopulation which expresses CCR6 in each GALT. These data indicate the possibility that class-switch recombination into IgG2b may occur at pre-GC B cells in SED of CePs in a similar mechanism to IgA class switching in PPs.

Previous studies have reported a decrease in the frequency of GC B cells and IgA^+^ B cells in PPs and the amount of IgA secreted in the intestinal contents of MyD88^−/−^ mice with a C57BL/6 background [15, 30]. Among the different cellular mechanisms in the GC responses of PPs, MyD88 signaling is important for regulating the development and function of Tfh cells and FDCs. Indeed, the expression of B cell activating factor and chemokine C-X-C motif ligand 13 by the FDCs in PP was reduced in MyD88^−/−^ mice [15]. In adoptive transfer experiments, stromal cell compartments containing FDCs from MyD88^−/−^ recipient mice were impaired in inducing GC B cells and IgA^+^ B cells in PPs. In addition, the lack of MyD88 signaling in CD4^+^ T cells was shown to decrease the induction of Tfh cells and GC responses in PPs, leading to a poor supply of IgA-producing cells in the lamina propria [14]. In contrast to these studies, we did not observe a remarkable reduction in GC B cells and IgA^+^ B cells in PPs or in the levels of IgA in the feces of MyD88^−/−^ mice on a BALB/c background. This contradiction might have resulted from the differences in mouse strains and/or in the intestinal microbiota across the facilities. Unlike IgA responses, our data indicate that the frequency of GC B cells and IgG2b^+^ B cells was decreased in the CePs of MyD88^−/−^ mice. Therefore, it is possible that the absence of MyD88 signaling leads to impaired function and/or decreased numbers of Tfh cells and FDCs, which may result in poor GC formation and class switching in B cells of CePs. In addition, we speculate that these immune and nonimmune cells in CePs and PPs may have different functions in the class switching of B cells. In future, it will be important to investigate the function of Tfh cells and FDCs in CePs, as opposed to that in the PPs. On the other hand, we could not rule out the possibility that some of differences observed between WT and MyD88^−/−^ mice might be due to the differences in the gut microbiota, because the mice used in the present study were not littermates or in cohousing conditions. Therefore, future studies will be needed to evaluate either condition.

PPs have important roles in generating high-affinity IgA against commensal bacteria in a T cell-dependent manner, wherein Tfh cells particularly play an important role. It has been reported that the excessive proliferation and dysfunction of Tfh cells due to the lack of the inhibitory receptor PD-1 induce changes in the affinity of IgA for the commensal bacteria and result in alterations in the composition of gut microbiota [9]. However, the binding capacity of the IgA generated by GALT in the large intestine has not been well investigated. In the present study, we showed that the GALT in the large intestine has a greater ability to produce microbiota-reactive IgA than the PPs, despite having fewer IgA^+^ B cells and producing lower amounts of total IgA in the cell culture. It is still unknown if in comparison to the PPs, CePs and CoPs have the ability to produce IgA with high affinity for commensal bacteria or conversely generate high levels of IgA with intermediate/low affinity for the commensal bacteria. Our data showed that the cells in PPs produced IgA that had lower capacity to bind commensal bacteria, a plausible explanation for which may be the presence of IgA that is specific for dietary antigens in addition to the commensal-specific IgA. Moreover, the previous study showed using PP-deficient mice that PPs were required for IgA which reacts to diet extracts [31].

Recently, it has been reported that IgG antibodies, particularly IgG2b and IgG3, bind to the commensal bacteria in the serum of healthy adult mice [18, 32]. These IgG antibodies have roles in protecting the host from systemic infections caused by bacteria, such as *Salmonella enterica* serovar Typhimurium [18] as well as in preventing the activation of mucosal helper T cells by limiting the translocation of commensal bacteria into mesenteric lymph nodes (MLN) [32]. Although it has been reported that IgG2b^+^ or IgG3^+^ B cells are more frequent in PPs and MLNs than in other lymphoid tissues [32], the primary site for the generation of these IgG isotype antibodies remains to be determined. Our data showed that IgG2b^+^ B cells are more abundant in CePs, and mononuclear cells from CePs produce high levels of total and microbiota-reactive IgG2b. Notably, the amount of microbiota-reactive IgG2b was lower in the serum of GF WT mice and SPF MyD88-deficient mice than in WT BALB/c mice with normal gut microbiota. Furthermore, this pattern was consistent with the levels of GC B cells and IgG2b^+^ B cells in CePs. Therefore, we anticipate that CePs may be an important source of the microbiota-reactive IgG2b in systemic circulation.

## 5. Conclusions

Taken together, our results provide evidence that CePs and PPs have different functions in the generation of class-switched antibody isotypes. Specifically, CePs produce a larger population of IgG2b^+^ B cells in a commensal microbiota-dependent manner. The IgG2b isotype antibodies produced by the mononuclear cells from CePs exhibited strong binding to commensal microbiota and were indicative of a potential source of microbiota-reactive IgG2b in systemic circulation. Thus, our data suggest that manipulating the mechanisms that mediate class switching via maintaining a well-balanced gut microbiota in the large intestine may contribute to the prevention of intestinal and systemic inflammation and infection. Hence, the contribution of the environmental factors and cellular mechanisms involved in antibody production in the large intestine needs to be further investigated.

## Data Availability

All data analyzed during this study are included in this manuscript and supplementary materials.

## Supplementary Materials

Supplementary Materials Figure S1: gating strategy of B cell subpopulations in GALT and their expression of IgA and IgG2b. Figure S2: the expression of CD38 and CCR6 on the B cell subpopulations separated by the expression of CD95 and GL7 in GALT from the small and large intestines in CV mice. Figure S3: the expression of IgM, IgG1, and IgG3 on B cells in GALT from the small and large intestines in CV and GF mice.
