# Supplementary material for: Cecal Patches Generate Abundant IgG2b-Bearing B Cells That Are Reactive to Commensal Microbiota
Source: J Immunol Res. 2022 May 4;2022:3974141. doi: 10.1155/2022/3974141 (PMC9095398; doi:10.1155/2022/3974141)
Supplement: Supplementary Materials — Figure S1: gating strategy of B cell subpopulations in GALT and their expression of IgA and IgG2b. Figure S2: the expression of CD38 and CCR6 on the B cell subpopulations separated by the expression of CD95 and GL7 in GALT from the small and large intestines in CV mice. Figure S3: the expression of IgM, IgG1, and IgG3 on B cells in GALT from the small and large intestines in CV and GF mice. [file 3974141.f1.pdf]

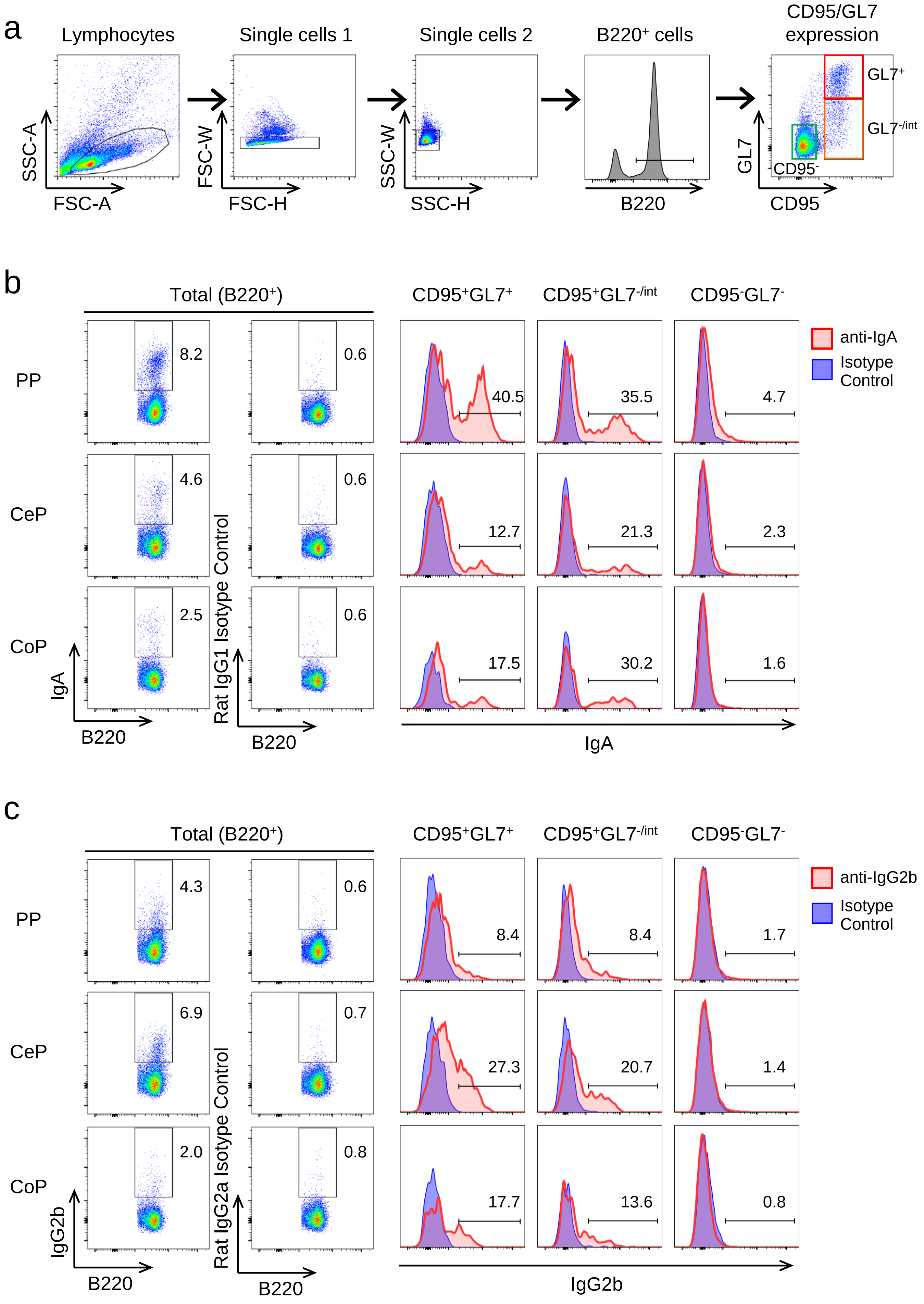
a
Lymphocytes
Single cells 1
Single cells 2
B220+ cells
CD95/GL7 expression
SSC-A
FSC-A
FSC-W
FSC-H
SSC-W
SSC-H
B220
GL7
CD95
GL7+
GL7-/int
CD95-
b
Total (B220+)
CD95+GL7+
CD95+GL7-/int
CD95-GL7-
anti-IgA
Isotype Control
PP
CeP
CoP
8.2
0.6
40.5
35.5
4.7
4.6
0.6
12.7
21.3
2.3
2.5
0.6
17.5
30.2
1.6
IgA
B220
Rat IgG1 Isotype Control
B220
IgA
c
Total (B220+)
CD95+GL7+
CD95+GL7-/int
CD95-GL7-
anti-IgG2b
Isotype Control
PP
CeP
CoP
4.3
0.6
8.4
8.4
1.7
6.9
0.7
27.3
20.7
1.4
2.0
0.8
17.7
13.6
0.8
IgG2b
B220
Rat IgG2a Isotype Control
B220
IgG2b

Figure.S1

Figure S1 Gating strategy of B cell subpopulations in GALT and their expression of IgA and IgG2b.

Flow cytometric analysis of mononuclear cells of GALT from CV mice was performed. (a) Gating strategy of B cell subpopulations separated by the expression of CD95 and GL7. (b, c) Flow cytometric analysis of the expression of IgA (b) or IgG2b (c) in $B220^{+}$ total B cells, $CD95^{+}GL7^{+}B220^{+}$ GC B cells, $CD95^{+}GL7^{-/int}B220^{+}$ pre-GC B cells and $CD95^{-}GL7^{-}B220^{+}$ naïve B cells. Data are representative of six to eight independent experiments.

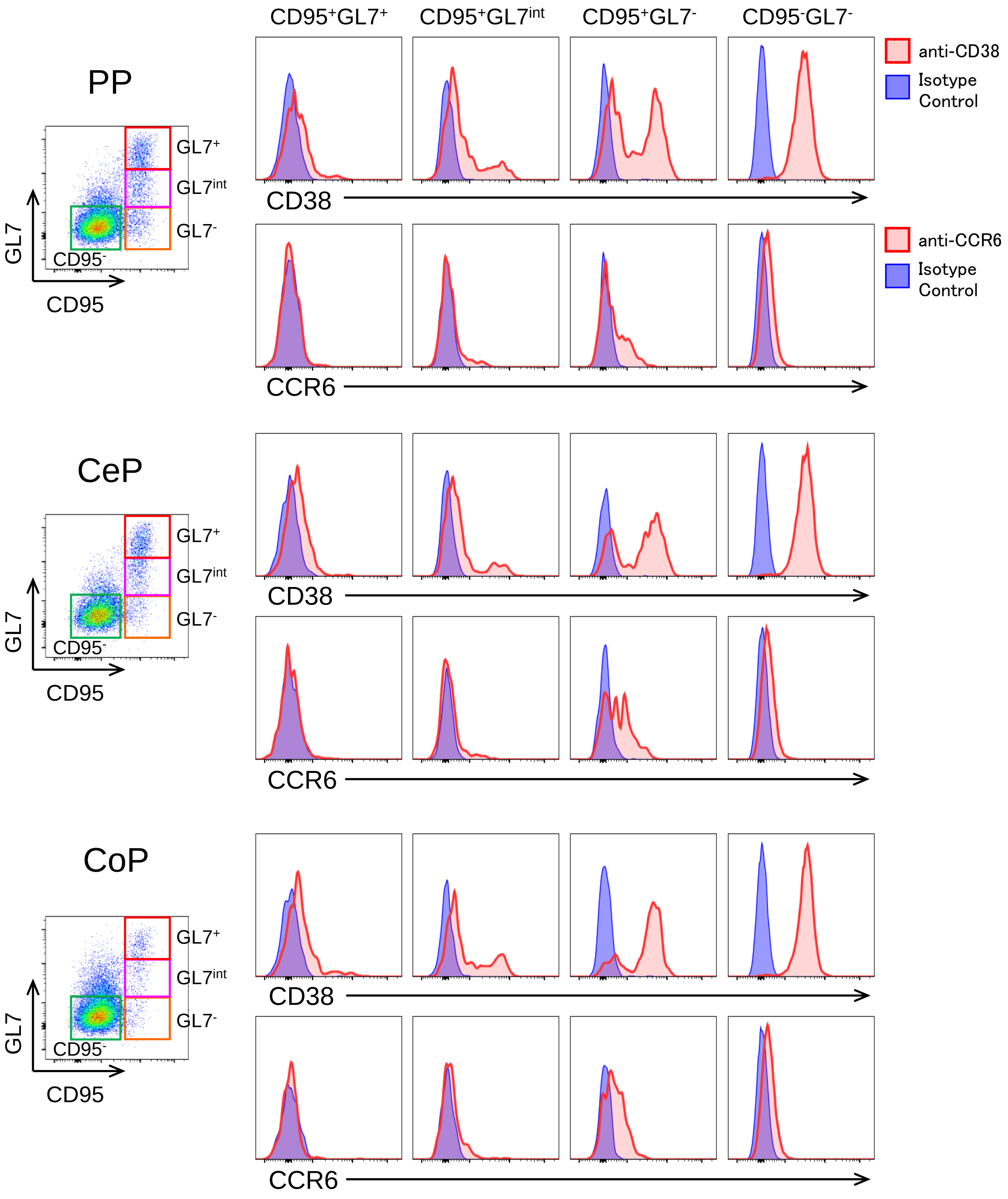

Figure S2 The expression of CD38 and CCR6 on the B cell subpopulations separated by the expression of CD95 and GL7 in GALT from small and large intestine in CV mice.

Flowcytometric analysis of mononuclear cells of GALT from CV mice was performed. Flow cytometric histogram plots of the expression of CD38 and CCR6 in $CD95^{+}GL7^{+}B220^{+}$, $CD95^{+}GL7^{int}B220^{+}$, $CD95^{+}GL7^{-}B220^{+}$, $CD95^{+}GL7^{-}B220^{+}$ cells. Data are representative of two independent experiments.

Figure.S2

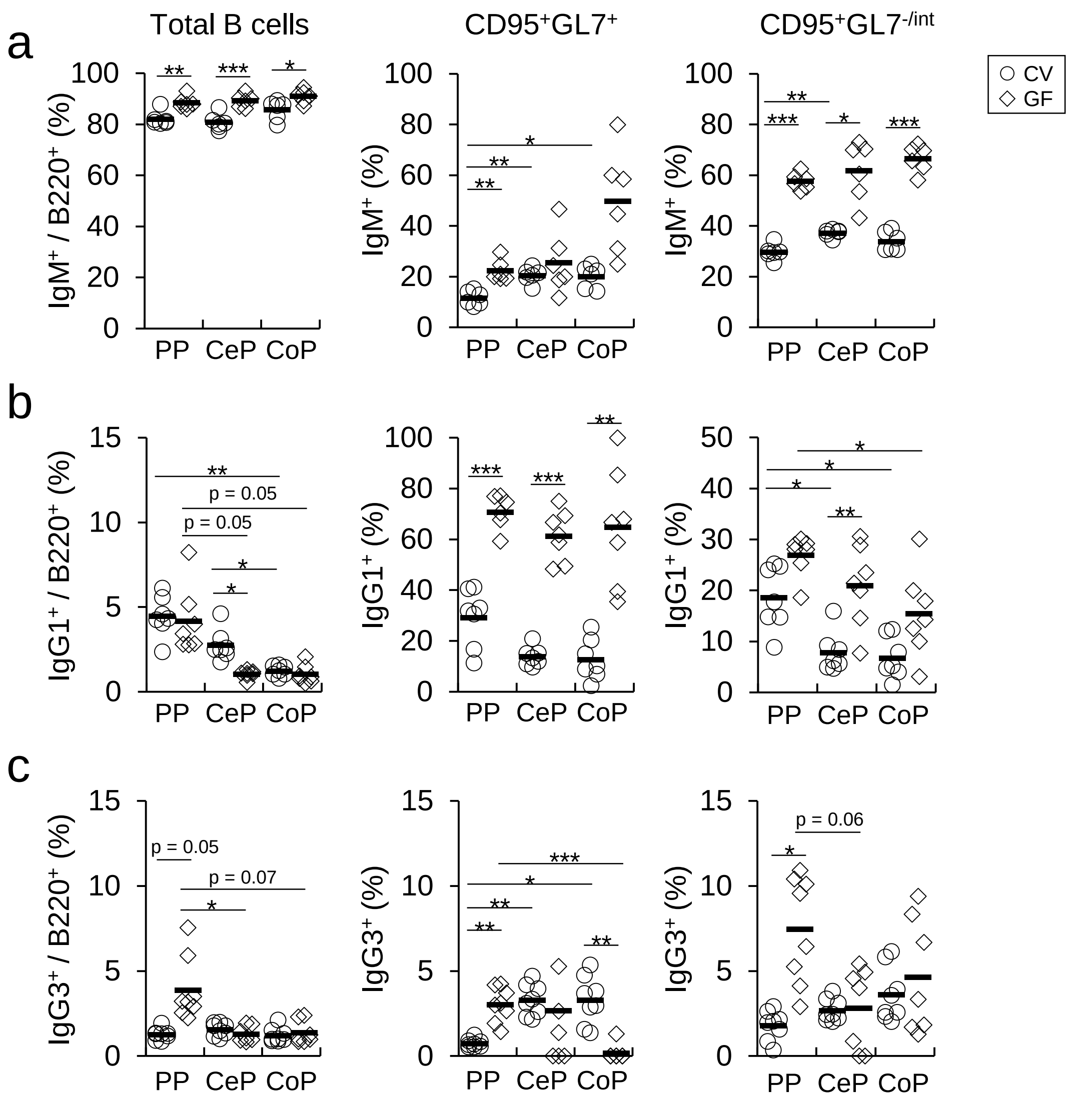

Figure S3 The expression of IgM, IgG1 and IgG3 on B cells in GALT from small and large intestine in CV and GF mice

Flowcytometric analysis of mononuclear cells of GALT from CV and GF mice was performed. The percentage of total ($B220^+$), $CD95^+GL7^+B220^+$ GC B cells, and $CD95^+GL7^{-/int}B220^+$ pre-GC B cells expressing IgM (a), IgG1(b) and IgG3(c). Data are shown as mean ± SD or representative FACS plot from six to eight independent experiments. *: $p < 0.05$, **: $p < 0.01$, ***: $p < 0.001$

Figure.S3
